# Oligoclonality of TRBC1 and TRBC2 in T cell lymphomas as mechanism of primary resistance to TRBC-directed CAR T cell therapies

**DOI:** 10.1038/s41467-025-56395-8

**Published:** 2025-01-29

**Authors:** Benjamin Thiele, Paul Schmidt-Barbo, Christoph Schultheiss, Edith Willscher, Thomas Weber, Mascha Binder

**Affiliations:** 1 Department of Biomedicine, Translational Immuno-Oncology, University and University Hospital Basel, Basel, Switzerland; 2 Division of Medical Oncology, University Hospital Basel, Basel, Switzerland; 3 Collaborative Research Institute Intelligent Oncology (CRIION), Freiburg, Germany; 4 Internal Medicine IV, Oncology/Hematology, Martin-Luther-University Halle-Wittenberg, Halle (Saale), Germany

**Keywords:** T-cell lymphoma, Translational immunology, VDJ recombination, Predictive markers, Cancer therapeutic resistance

**arising from** M. Ferrari et al. *Nature Communications* 10.1038/s41467-024-45854-3 (2024)

With great interest, we have read the recent publication of Ferrari and Righi et al. which showcases the precise engineering of immunotherapies against the T cell receptor β-chain constant domain 2 (TRBC2) in T cell lymphomas^1^. A major challenge in targeting T cell lymphomas is the absence of a unique marker to separate healthy from malignant T cells, avoiding live-threatening T cell depletion and CAR T cell fratricide. The distinction between TRBC1 and TRBC2, differing by only two amino acids at the accessible epitope, is a remarkable achievement complementing the group’s success in targeting TRBC1^2^. TRBC1-specific CAR T cell therapy is under clinical evaluation (NCT03590574). Promising phase I data show early disease progression in 2 of 4 patients treated at the target dose^3^. The expansion of their research to target TRBC2 is a commendable advancement with the potential to treat all patients with T cell receptor β-chain expressing lymphomas. However, the observed early progression of patients raises suspicion that limited expansion and persistence of transfused CAR T cells may not be the only explanation for insufficient tumor control^3^. Mechanisms of primary resistance might be at work which warrant further investigation.

Genomic sequencing studies, particularly in solid tumors, challenge the linear tumor evolution model, favoring a branching precursor cell evolution leading to a complex cellular landscape^4^. Lymphomas have traditionally been viewed as neoplasms that emerge from mature cells. This was due to their immunophenotype that closely resembles subsets of normal differentiated T cells and their rearranged T cell receptor V(D)J genes. Yet, recent sequencing studies reveal genomic complexity in driver genes and clonal hematopoiesis, suggesting malignant transformation originates in immature precursor cells - a finding supported by T cell receptor clonality studies^5–7^. While monoclonality for the TRG locus - which undergoes recombination early in T cell development - is found in most T cell lymphomas, the V(D)J-rearranged TRB and TRA loci are often oligoclonal. Together, these findings suggest that malignant transformation may often be initiated at the stage of a lymphoid precursor cell before TRB and TRA recombination rather than in a mature post-thymic T cell as illustrated in Fig. 1a^8,9^. This clonal heterogeneity certainly contributes to poor or non-durable treatment outcomes. Importantly, the observed oligoclonality for the TRB locus in many T cell lymphomas extends across different TRB J-genes which suggests that heterogeneous TRBC segments may coexist within such oligoclonal lymphomas. This is particularly evident when TRB J1 and J2 family genes, which are linked to TRBC1 and 2 expression, are utilized by different subclones. To our knowledge, there was no study explicitly looking for TRBC oligoclonality in T cell lymphomas so far.

**Fig. 1:**
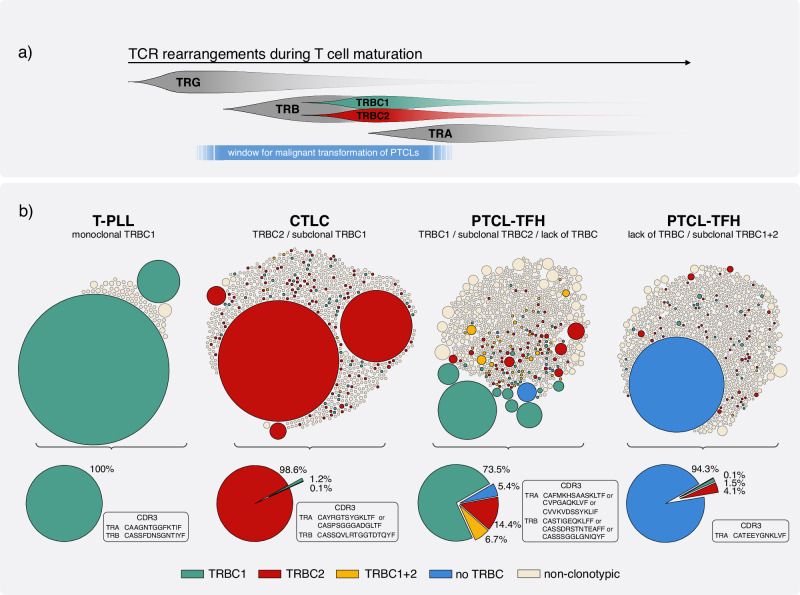
TCR maturation and TRBC clonality in selected peripheral T cell lymphoma cases. **a** illustrates TRBC clonality in relation to the maturation stage of the lymphoma cell of origin. In panel **b** four characteristic cases of peripheral T cell lymphomas are highlighted with single-cell TRBC expression plots of sequenced T cells. Malignant T cells, defined by their listed clonotypic TRA or TRB CDR3 rearrangements, are colored according to their TRBC expression with bubble sizes normalized to the case-specific single cell read counts. Detailed definition of the clonotypic CDR3 rearrangements are listed in the “methods” section.

Since oligoclonality for TRBC1 and TRBC2 would represent a major obstacle to achieve long term remissions with TRBC1 or TRBC2 directed CAR T cell therapy, we set out to analyze T cell receptor single cell sequencing data of CD3+ sorted T cells from twelve distinct T cell lymphoma cases for TRBC expression. To increase validity, we used data from different sources for this analysis: Two cases were single-cell sequenced by our own group and the remaining cases were published single-cell datasets from two separate groups (Liu et al.^5^ and Suma et al.^6^). We assessed distinct types of peripheral T cell lymphomas including one case of T-prolymphocytic leukemia (T-PLL), one case of primary cutaneous T cell lymphoma (CTCL), and ten cases of nodal T-follicular helper cell lymphoma (PTCL-TFH). To ensure that our analysis was not skewed by the presence of non-malignant bystander cells exhibiting diverse TRBC usage, we applied a stringent definition of clonotypic cells by using clonotypic TRA / TRB complementarity-determining region 3 (CDR3) as molecular barcodes (see “Methods”). One fourth (3/12) of these unselected cases fulfilled the paradigm of dichotomy of TRBC1 or TRBC2 expression by exclusively expressing one of the chains in the main tumor clone and its subclones. In two cases this was the TRBC2 chain and in one case, displayed in Fig. 1b, the TRBC1 chain. In seven cases, heterogeneic TRA/TRB pairings resulted in a fraction of malignant cells expressing the alternative TRBC chain that was not used by the major lymphoma clone exemplified by three cases with discordant tumor subclones labeled in red and green in Fig. 1b. In addition, there were two cases in which the major malignant clone completely lacked TRBC expression which is illustrated by a case with a dominant blue clone in Fig. 1b.

In conclusion, our analysis of twelve unselected peripheral T cell lymphomas revealed TRBC oligoclonality, suggesting malignant transformation often precedes TRB rearrangement, as depicted in Fig. 1a^8,9^. Consequently, our results challenge the binary assumption of restricted TRBC1, or TRBC2 expression of peripheral T cell lymphomas proposed by Ferrari and Righi et al.^1^. This binary view on TRBC expression is fundamental to TRBC-directed therapies, yet the employed screening methods in the ongoing clinical trial may lack the sensitivity to detect subclonal TRBC heterogeneity^3^. With certain cases exhibiting clonal or subclonal loss of TRBC expression, our findings also align with prior studies, such as those by Liu et al., who documented complete loss of T cell receptor expression in 5 of 11 patients with primary cutaneous T cell lymphoma^5^. Furthermore, our results are consistent with a recent flow cytometry investigation on T cell lymphomas with specific TRBC1 and TRBC2 directed antibodies. Notably, across multiple flow cytometry charts with expert gating for malignancy, there was a consistent presence of a subpopulation that exhibited differing TRBC expression, thus validating our single-cell expression-based findings by a distinct phenotypic method (see Horna et al. figure 6a, b, d–f)^10^. Therefore, both clonal heterogeneity for TRBC1 and TRBC2 usage, as well as loss of T cell receptor expression might turn out to be potential mechanisms of primary treatment failure in patients undergoing TRBC1 or TRBC2 directed CAR T cell therapy. This note of caution also applies for therapies targeting a specific TRB V-gene, a potential alternative treatment strategy for T cell lymphoma recently reported by Ren et al.^11^.

Our study has limitations, but its strength and novelty lie in its specific focus on TRBC heterogeneity within the malignant clonotypic compartment, with results that may differ from studies employing integrated broader transcriptomic and genomic data. Malignant clones were defined by a subjective clonality threshold (> 5%), consistent with prior studies^12–15^, but stricter than others (e.g., > 1.5%, Suma et al.^6^). However, poor RNA/DNA quality may always affect TCR sequencing accuracy, leading to clonotype frequency misestimation. Combining TCR and mutational data could improve accuracy but faces technical limitations, particularly for low abundance clonotypes. Although our single-cell analysis of twelve cases cannot estimate the fraction with differing or absent TRBC expression, indirect evidence from J-gene usage data by Iyer et al. and our work suggests TRBC oligoclonality or subclonal loss in up to 50% of T cell lymphoma patients^8,9^. Comprehensive clonality profiling will therefore be mandatory for effective allocation of these complex cellular therapies. Further analysis of patients progressing on TRBC1 directed CAR T cell therapy will elucidate if this remarkable endeavor of precision medicine will remain a tilting at windmills or if the hydra could - at least in some cases - be slayed.

## Methods

### Patient sampling

Written informed consent was obtained from each patient for the use of their biological material as approved by the ethics commissions in Hamburg (PV3400, PV4767) and Halle, Germany (2014-075) and in line with the Helsinki Declaration of 1975, as revised in 2000. PBMCs of a patient with T-PLL and a patient with PTCL-TFH were isolated by density gradient centrifugation and cryopreserved in FCS + 10% DMSO (v/v).

### Single-cell TCR sequencing

Single-cell TCR sequencing was performed using the Next GEM Single Cell 5’ Kit v2 and Chromium Single Cell Human TCR Amplification kits (10X Genomics, Pleasanton, CA, USA). CD3 T cells were FACS-sorted (CD3-FITC, clone SK7, BD Biosciences) from cryopreserved PBMCs and processed on a 10X Chromium Controller (10X Genomics). TCR libraries were quality checked on an Agilent Bioanalyzer and sequenced on an Illumina NovaSeq 6000 system (S4 flow cell) with 150 base pairs and paired-end configurations. Additional CTCL and PTCL-TFH single-cell TCR sequencing data was obtained from the referenced repositories by Suma et al. and Liu et al.^5,6^. A CTCL case from the Liu et al. data was selected to illustrate the potential applicability of our findings to this specific WHO lymphoma entity, which is not included in the ongoing CAR T cell trial.

### Data processing, analysis, and plotting

Single-cell TCR sequencing data was processed with the Cell Ranger V(D)J pipeline (v7.1.0) and aligned to the vdj-GRCh38 reference genome. Only productive rearrangements were used for downstream analysis. Based on published clonality ranges/thresholds^12–15^, TRA or TRB CDR3s were defined as clonotypic if constituting > 5% of the clonal space in the respective TRA or TRB immune repertoire. Malignant cells were defined by the presence of at least one clonotypic CDR3 sequence and analyzed for TRBC1 or TRBC2 expression. We discarded cells with missing TRBC expression if the fraction of those cells was less than one third of all cells sharing the same clonotypic CDR3 sequence. This way we eliminated potential technical artifacts and low sequencing coverage of the J-TRBC region. All analyzes were performed using python (v3.11.6). Bubble plots were generated using pandas (v2.1.4), NumPy (v1.26.4) and matplotlib (v3.8.0). Illustrations and final figure arrangements were done in Adobe Illustrator (v28.1).

### Reporting summary

Further information on research design is available in the Nature Portfolio Reporting Summary linked to this article.

## Supplementary information

Reporting Summary

## Source data

Source Data

## Data Availability

The single-cell TCR sequencing generated in this study have been deposited in the European Nucleotide Archive under accession number PRJEB74284. The data of Suma et al. and Liu et al. are available on the European Genome-Phenome Archive and the Genome Sequence Archive for Human, respectively. Access requires approval as detailed in their publications^5,6^. Processed data for Fig. 1 are provided in the accompanying source data file.
