# Supplementary material for: Oligoclonality of TRBC1 and TRBC2 in T cell lymphomas as mechanism of primary resistance to TRBC-directed CAR T cell therapies
Source: Nat Commun. 2025 Jan 29;16:1104. doi: 10.1038/s41467-025-56395-8 (PMC11779876; doi:10.1038/s41467-025-56395-8)
Supplement: Supplementary file 1 — Reporting Summary [file 41467_2025_56395_MOESM1_ESM.pdf]

nature portfolio

Corresponding author(s): Prof. Dr. Mascha Binder
University Hospital Basel
Petersgraben 4, 4031 Basel Switzerland

Last updated by author(s): Jan 1, 2025

Reporting Summary

Nature Portfolio wishes to improve the reproducibility of the work that we publish. This form provides structure for consistency and transparency in reporting. For further information on Nature Portfolio policies, see our Editorial Policies and the Editorial Policy Checklist.

Statistics

For all statistical analyses, confirm that the following items are present in the figure legend, table legend, main text, or Methods section.

n/a	Confirmed	
☐	☒	The exact sample size (*n*) for each experimental group/condition, given as a discrete number and unit of measurement
☐	☒	A statement on whether measurements were taken from distinct samples or whether the same sample was measured repeatedly
☒	☐	The statistical test(s) used AND whether they are one- or two-sided<br>*Only common tests should be described solely by name; describe more complex techniques in the Methods section.*
☒	☐	A description of all covariates tested
☒	☐	A description of any assumptions or corrections, such as tests of normality and adjustment for multiple comparisons
☒	☐	A full description of the statistical parameters including central tendency (e.g. means) or other basic estimates (e.g. regression coefficient) AND variation (e.g. standard deviation) or associated estimates of uncertainty (e.g. confidence intervals)
☒	☐	For null hypothesis testing, the test statistic (e.g. *F*, *t*, *r*) with confidence intervals, effect sizes, degrees of freedom and *P* value noted<br>*Give P values as exact values whenever suitable.*
☒	☐	For Bayesian analysis, information on the choice of priors and Markov chain Monte Carlo settings
☒	☐	For hierarchical and complex designs, identification of the appropriate level for tests and full reporting of outcomes
☒	☐	Estimates of effect sizes (e.g. Cohen's *d*, Pearson's *r*), indicating how they were calculated

Our web collection on statistics for biologists contains articles on many of the points above.

Software and code

Policy information about availability of computer code

Data collection	Illumina MiSeq Software (v4.1.0)
Data analysis	Cell Ranger V(D)J (v7.1.0; alignment to vdj-GRCh38), python (v3.11.6), pandas (v2.1.4), NumPy (v1.26.4), matplotlib (v3.8.0), Adobe Illustrator (v28.1)

For manuscripts utilizing custom algorithms or software that are central to the research but not yet described in published literature, software must be made available to editors and reviewers. We strongly encourage code deposition in a community repository (e.g. GitHub). See the Nature Portfolio guidelines for submitting code & software for further information.

Data

Policy information about availability of data

All manuscripts must include a data availability statement. This statement should provide the following information, where applicable:

- Accession codes, unique identifiers, or web links for publicly available datasets
- A description of any restrictions on data availability
- For clinical datasets or third party data, please ensure that the statement adheres to our policy

Single-cell TCR sequencing data has been deposited at the European Nucleotide Archive (accession number: PRJEB74284). For data of Suma et al. and Liu et al. please refer to their respective publications 6,7.

Research involving human participants, their data, or biological material

Policy information about studies with human participants or human data. See also policy information about sex, gender (identity/presentation), and sexual orientation and race, ethnicity and racism.

Reporting on sex and gender	Neither sex nor gender was the subject of this investigation.
Reporting on race, ethnicity, or other socially relevant groupings	Neither race nor ethnicity nor other socially relevant groups were the subject of this investigation.
Population characteristics	Population characteristics are not relevant to this investigation.
Recruitment	There were no specific inclusion or exclusion criteria for this investigation. One patient with T-PLL and one patient with PTCL-TFH consented to donate blood. There is no relevant bias for this investigation.
Ethics oversight	Ethics commissions in Hamburg (PV3400, PV4767) and Halle, Germany (2014-075) approved the investigation.

Note that full information on the approval of the study protocol must also be provided in the manuscript.

Field-specific reporting

Please select the one below that is the best fit for your research. If you are not sure, read the appropriate sections before making your selection.

☒ Life sciences ☐ Behavioural & social sciences ☐ Ecological, evolutionary & environmental sciences

For a reference copy of the document with all sections, see nature.com/documents/nr-reporting-summary-flat.pdf

Life sciences study design

All studies must disclose on these points even when the disclosure is negative.

Sample size	Overall 12 samples were analyzed. Two cases were single-cell sequenced by our own group and the remaining cases were published single-cell datasets from two separate groups (Liu et al. and Suma et al.). No sample size calculation was performed for this investigation since qualitative evidence for heterogeneity over a diverse set of T-cell lymphomas was our main research focus.
Data exclusions	No data was excluded from this analysis.
Replication	Reproducibilty of the reported findings is reinforced by the integration of data from three distinct sources: our primary dataset and two external, independently collected datasets. All data utilized in this research is accessible in public repositories, ensuring full transparency and reproducibility.
Randomization	Randomization was not relevant to this investigation since our main focus was the qualitative description of T-cell lymphoma heterogeneity.
Blinding	Blinding was not relevant to this investigation since our main focus was the qualitative description of T-cell lymphoma heterogeneity.

Reporting for specific materials, systems and methods

We require information from authors about some types of materials, experimental systems and methods used in many studies. Here, indicate whether each material, system or method listed is relevant to your study. If you are not sure if a list item applies to your research, read the appropriate section before selecting a response.

Materials & experimental systems

n/a	Involved in the study	
☒	☐	Antibodies
☒	☐	Eukaryotic cell lines
☒	☐	Palaeontology and archaeology
☒	☐	Animals and other organisms
☒	☐	Clinical data
☒	☐	Dual use research of concern
☒	☐	Plants

Methods

n/a	Involved in the study	
☒	☐	ChIP-seq
☒	☐	Flow cytometry
☒	☐	MRI-based neuroimaging

Plants

Seed stocks	*Report on the source of all seed stocks or other plant material used. If applicable, state the seed stock centre and catalogue number. If plant specimens were collected from the field, describe the collection location, date and sampling procedures.*
Novel plant genotypes	*Describe the methods by which all novel plant genotypes were produced. This includes those generated by transgenic approaches, gene editing, chemical/radiation-based mutagenesis and hybridization. For transgenic lines, describe the transformation method, the number of independent lines analyzed and the generation upon which experiments were performed. For gene-edited lines, describe the editor used, the endogenous sequence targeted for editing, the targeting guide RNA sequence (if applicable) and how the editor was applied.*
Authentication	*Describe any authentication procedures for each seed stock used or novel genotype generated. Describe any experiments used to assess the effect of a mutation and, where applicable, how potential secondary effects (e.g. second site T-DNA insertions, mosiacism, off-target gene editing) were examined.*
